# LTPConstraint: a transfer learning based end-to-end method for RNA secondary structure prediction

**DOI:** 10.1186/s12859-022-04847-z

**Published:** 2022-08-23

**Authors:** Yinchao Fei, Hao Zhang, Yili Wang, Zhen Liu, Yuanning Liu

**Affiliations:** 1 College of Computer Science and Technology, Jilin University, Changchun, China; 2 Key Laboratory of Symbolic Computation and Knowledge Engineering, Ministry of Education, Jilin University, Changchun, China; 3 Graduate School of Engineering, Nagasaki Institute of Applied Science, Nagasaki, Japan

**Keywords:** RNA secondary structure, Bi-LSTM, Transformer, Generator, Transfer learning

## Abstract

**Background:**

RNA secondary structure is very important for deciphering cell’s activity and disease occurrence. The first method which was used by the academics to predict this structure is biological experiment, But this method is too expensive, causing the promotion to be affected. Then, computing methods emerged, which has good efficiency and low cost. However, the accuracy of computing methods are not satisfactory. Many machine learning methods have also been applied to this area, but the accuracy has not improved significantly. Deep learning has matured and achieves great success in many areas such as computer vision and natural language processing. It uses neural network which is a kind of structure that has good functionality and versatility, but its effect is highly correlated with the quantity and quality of the data. At present, there is no model with high accuracy, low data dependence and high convenience in predicting RNA secondary structure.

**Results:**

This paper designs a neural network called LTPConstraint to predict RNA secondary structure. The network is based on many network structure such as Bidirectional LSTM, Transformer and generator. It also uses transfer learning to train modelso that the data dependence can be reduced.

**Conclusions:**

LTPConstraint has achieved high accuracy in RNA secondary structure prediction. Compared with the previous methods, the accuracy improves obviously both in predicting the structure with pseudoknot and the structure without pseudoknot. At the same time, LTPConstraint is easy to operate and can achieve result very quickly.

## Background

**Fig. 1:**
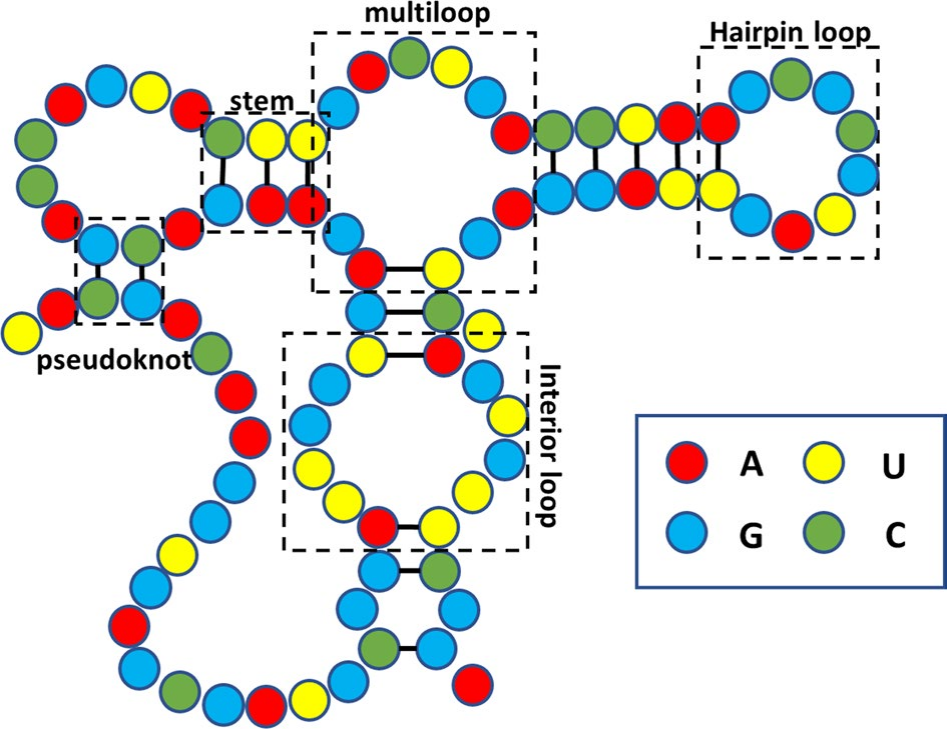
RNA secondary structure. The red, yellow, blue, and green spheres represent adenine, guanine, cytosine, and uracil, respectively. Legends of common structures in RNA secondary structures such as multiloop and stem are marked in the figure

Ribonucleic Acid (RNA) is a carrier of life genetic information. The regular activities of living organisms depend on the correct expression of coding RNA (such as tRNA, mRNA) and non-coding RNA [1]. It acts on all processes of cell activity. It directly or indirectly relates to the regulation and occurrence of diseases [2]. RNA is a long-chain-like molecule composed. It is usually composed of four kinds of bases which are connected by phosphoric diester bond. Hydrogen bonds can also be formed between bases and such two bases connected by Hydrogen bonds are called a pair. Pairs can be divided into canonical pair and non-canonical pair. The canonical pair refers to the pairing of AU, GC, and GU, while the non-canonical pair is a pairing style other than above [3]. RNA has a quaternary structure academically. The primary structure of RNA is a single strand composed of base pairs. The secondary structure of RNA is a hairpin-shaped composite structure formed by convolutional folding of the primary structure of RNA. The tertiary structure of RNA is a spatial structure formed by further bending the spiral based on the secondary structure. The quaternary structure of RNA is a mixture of nucleic acid and protein produced by the interaction of RNA and protein. As we can see in the Fig. 1, the secondary structure of RNA forms various structure after helical folding, including hairpin loop, stem, interior loop and pseudoknot.

**Fig. 2:**
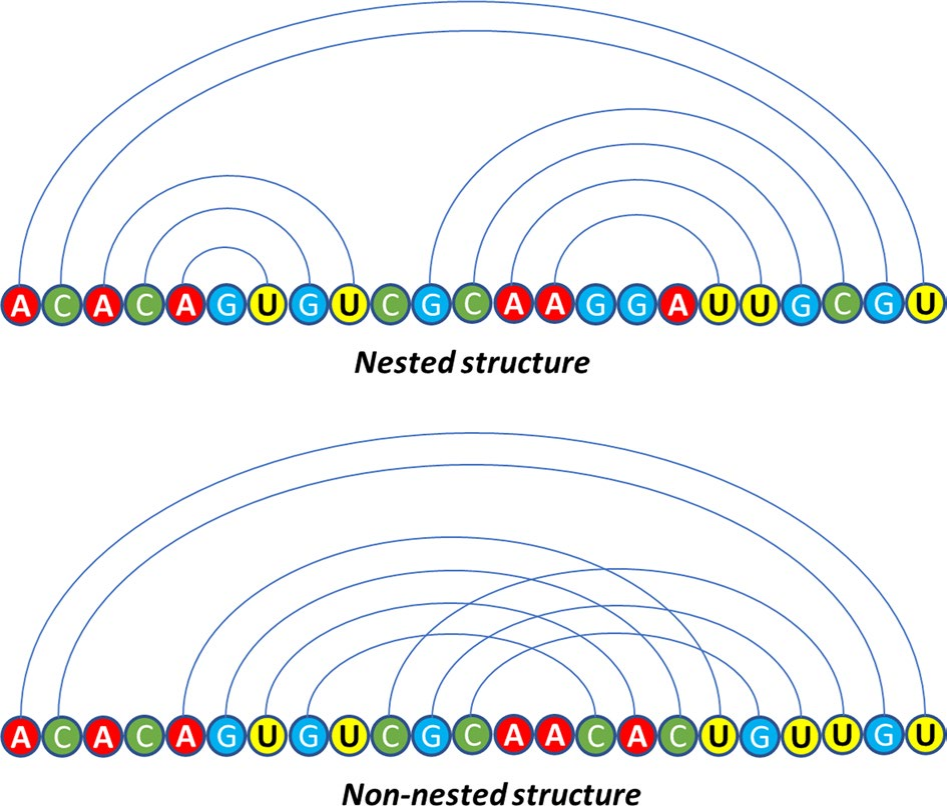
Nested and non-nested structure. The diagram above represents a nested structure and the diagram below represents a non-nested structure

Pseudoknot generally appears in the pair of single-stranded ring surrounded by the stem [4]. This structure is different from some of the above planar structure in that it is related to the spatial structure of RNA. The prediction of pseudoknot is of great importance because pseudoknot has an important influence on the life activities involved by RNA. However, the secondary structure containing pseudoknot will form a non-nested structure from a planar view, as we can see in the Fig. 2. All possible nested structure can be quickly obtained by using dynamic programming algorithms, but the secondary structure contains pseudoknot can’t, so it is difficulty to predict the secondary structure containing pseudoknot [5].

The tertiary structure of RNA is a key to interpreting the relationship between RNA’s structure and function, especially the structure called noncoding RNA [6]. Meanwhile, the tertiary structure of RNA is also the most direct material to analysing the state of RNA that is difficult to characterize [7]. Generally speaking, the secondary structure tends to be formed quicker than tertiary structure [8], so before predicting the tertiary structure of RNA, obtaining accurate secondary structure is the basis. Also, the secondary structure of RNA is related to RNA’s function [9]. Therefore, accurately predicting secondary structure is vital for studying RNA.

Scholars tried many methods from different fields to predict secondary structure. Initially, they obtained RNA secondary structure from biological experiments. DMS-MaPseq is a robust assay method that uses the advantages of dimethyl sulfate (DMS)-mutational profiling and sequencing (MaPseq), making it easy to modify RNA in vitro, in cells, and virions [10], thereby enabling Determine the various levels of RNA structure. SHAPE [11](Selective 2’-hydroxyl acylation analyzed by primer extension) method can be used to analyze selective 2’-hydroxyl acylation reactions in living cells by primer extension, and the High-throughput data of relevant nucleotides in paired or unpaired state can be obtained with a single base-pair resolution by SHAPE [12]. In addition to the above methods, the most commonly used experimental method is the X-ray crystallography and nuclear magnetic resonance [13]. Both methods can also provide structural information with a single base pair resolution. In summary, these experimental methods have two common characteristics. They are high cost and low yield. These performances make it inefficient when predicting a large number of RNA sequences and make experimental methods hard to be used on a large scale.

To reduce the cost of prediction and improve efficiency. Academics has turned to computing methods to predict RNA secondary structure. The computing methods can be divided into two types, comparative sequence analysis and folding algorithms using thermodynamic, statistical or probabilistic scoring schemes [14]. Comparative sequence analysis [15] predicts the secondary structure of RNA by using the conservative base pairs between the homologous sequences [16]. This method is highly accurate if homologous sequences can be obtained, but there are only a few known RNA families causing not enough data, affecting this method to be promated. The folding algorithm often divides the entire sequence into sub-blocks. It then generates the optimal secondary structure after scoring each sub-block based on thermodynamic principles or scoring schemes such as statistics and probability. The representative one is the minimum free energy model using a dynamic programming algorithm [17]. Its implementation include RNAstructure and RNAfold. According to the principle of minimum free energy algorithm, RNAstructure [18] uses Zuker algorithm [19] to obtain the optimal secondary structure. The biggest advantage of this software is that it adds many additional modules to extend the function of Zuker algorithm, enriching the user experience, and its graphical interface makes users operate conveniently. The thermodynamic data provided by Turner was used to calculate the free energy of each substructure [20]. RNAfold [21] also uses the free energy parameters. The minimum free energy method has an upper limit of accuracy. This is because many real RNA secondary structure are not necessarily the structure with the minimum free energy, which leads to the assumption of the minimum free energy method cannot always hold.

Other computing methods use machine learning. CONTRAfold [22] uses stochastic context-free grammar (SCFG). SCFG model parameters are derived using an automatic statistical learning algorithm. This is a big innovation, but even the best SCFG model doesn’t perform so well as the method of minimum free energy model. In [23], the author successfully combined deep learning with the thermodynamic nearest-neighbor model. According to the relationship between SHAPE data and state inference, bidirectional LSTM was used to extract sequence features, and then the state inference of the sequence was obtained through classifier based on these features. The SHAPE value is obtained according to the relation formula between SHPAE data and state inference. Then, the calculated SHAPE value was used as a soft constraint for a recent thermodynamic model called GTfold [24]. This method achieved high accuracy according to the author’s description. However this method is still not a complete end-to-end RNA secondary structure prediction method. Also, this method predict RNA secondary structure based on GTfold, and the SHAPE value only serves as supplementary information to improve the prediction accuracy of GTfold.

In recent years, deep learning has achieved breakthrough in computer vision and natural language processing. On image translation, A model called Pix2pix makes this kind of problem obtain a general and good enough solution. The traditional method of image translation only uses an original CNN model to minimize the Euclidean distance between the prediction and target without a good loss, and the result can only get a fuzzy output [25]. Therefore, traditional models often require mannually designing precise loss functions to guide CNN to complete tasks. Pix2pix [26] model is designed based on the basis of GAN [27]. It makes the output indistinguishable from reality by optimizing a high-dimensional problem. To be specific, Pix2pix constructed a generator with strong feature extraction ability and a discriminator that scores the difference between input and output, making this structure a universal method in image translation [28, 29].

**Fig. 3:**
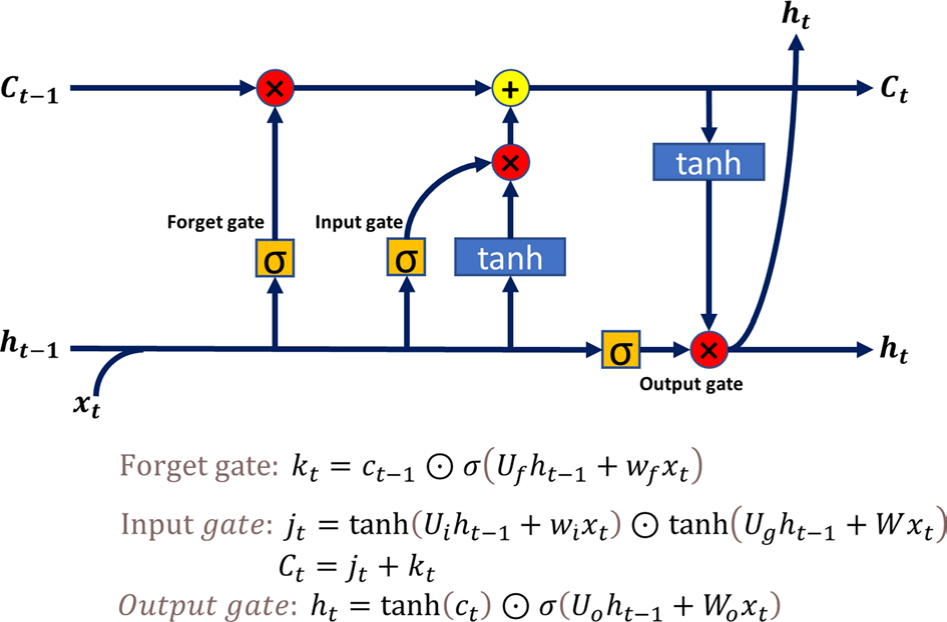
The structure of LSTM. The diagram shows a basic unit of the LSTM. Each time the data passes through three gates, it will get a cell state, a hidden state and an output state of this moment. The hidden state will be passed to the next moment for calculation, while the output state will be directly output

LSTM and Transformer are two excellent structure that have emerged in natural language processing. LSTM(Long Short-Term Memory) [30] is the most commonly used model structure for processing indefinite length linear sequences. It is improved from RNN [31] structure. As can be seen in the Fig. 3 In LSTM, three gate structure called forgetting gate, information enhancement gate, and output gate are added. The LSTM’s three-gate structure enhances RNN’s ability to extract features over long distances. By overlaying network structure to increase the depth of information processing, LSTM can handle almost all semantic problems using an encoder-decoder [32] framework conbining with the Attention [33] mechanism [34].

**Fig. 4:**
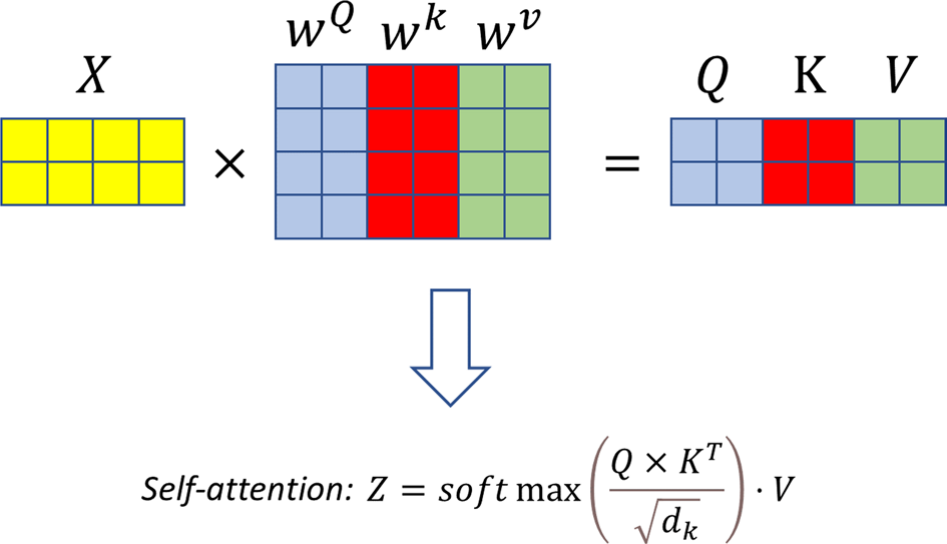
The mechanism of self-attention. The graph shows a numerical transformation of self-attention. Three vectors of Query, Key and Value are obtained from the input X according to different weights, and then these three vectors are put into the following formula to obtain self-attention

Transformer [35] is one of the most notable achievements of deep learning in recent years. Transformer has achieved breakthrough achievements in several areas, especially in natural language processing [36] and computer vision [37, 38]. It cleverly uses self-attention or multi-head self-attention for semantic extraction. As can be seen from the Fig. 4, Transformer is a structure similar to full connection, which can extract the connection between each word in a sentence. Its mult-head self-attention can focus on different positions in a sentence, so as to better extract semantics. Transformer operates on all words of the entire sentence at the same time, rather than processing each word sequentially, which brings strong parallel computing to Transformer. Similar to LSTM, Transformer can be nested into an encoder-decoder model to accomplish various semantic tasks, and its performance in many tasks is even better than the model using LSTM.

Both LSTM and Transformer have their strengths and weaknesses. In [39], the author compares LSTM with Transformer in terms of semantic feature extraction ability, long-distance capture ability, task comprehensive feature extraction ability and parallel computing ability. The results show that Transformer is better than LSTM in four aspects, especially in terms of parallel computing capability. As LSTM is a time-sequence linear structure, its parallel computing capability is very weak, which is a structural defect that is difficult to make up for. Moreover, the transformer structure solves the problem of long memory loss that can still occur in LSTM when the sequence is too long [40]. However, the self-attention mechanism lacks modeling of time dimension. In other words, Transformer is not sensitive to the word order of input statements. As a result, the position encoding mechanism is used in the current Transformer structure. It adds sequential timing data into Transformer directly. Of course, such a mechanism is not as good as the natural temporal structure of LSTM. This is obviously a stopgap [41], and this can result in the Transformer not performing well on word order sensitive tasks. Although LSTM is suitable for the above scenarios, it is incapable of training in the face of large data sets. Its lack of parallel computing time series structure leads to slow operation. At the same time, when the amount of learned data exceeds a certain threshold, LSTM can no longer be improved. Transformer can handle such scenarios very well according the description above.

With the development of neural network, the depth of the model is getting larger and larger, And the amount of data required to fit the network also increases. Nowadays, it has become a time-consuming and laborious task to train a model from scratch [42]. To solve this problem, the transfer learning of deep neural networks was born [43]. The core of transfer learning [44] is to use the pre-trained model. Pre-trained model [45] was obtained by training some network structure with high robustness using high quality data set. Pre-trained model can then be trasfered to train other relevant data. In other words, There is no need to train a model from scratch for a specific problem. We can find a pre-trained model of similar problems and then train it with a small amount of problem-specific data, it will significantly reduce the training time and the amount of data that required to fit because the pre-trained model has learned much relevant feature during pre-training, so the more features pre-training data shares with the problem-specific data, the easier the transfer learning process will be. We just need to design the fine-tuning mechanism [46]. The operation is simple and easy to understand, but the effect is significant [47].

The prediction of RNA secondary structure depends on the data of biochemistry experiment for a long time, which affects the progress of the research on this problem. We believe that we can create a model based on deep learning, and then correct the output of our model by taking some thermodynamic or biological research results of RNA secondary structure as prior knowledge, so the efficiency and accuracy of this model will be significantly improved. At the same time, the neural network model learns the structural features completely according to the input data, so we believe that if we have enough RNA structure containing pseudoknot, the feature of pseudoknot can also be learned by our network, so as to make up for the shortcomings of the past methods in predicting pseudoknot.

## Methods and materials

In this section, we will describe the structure of LTPConstraint according to the idea and the correctness analysis of LTPConstraint. We will also describe the transfer learning method used by LTPConstraint and the data set after processing.

### Methods

**Fig. 5:**
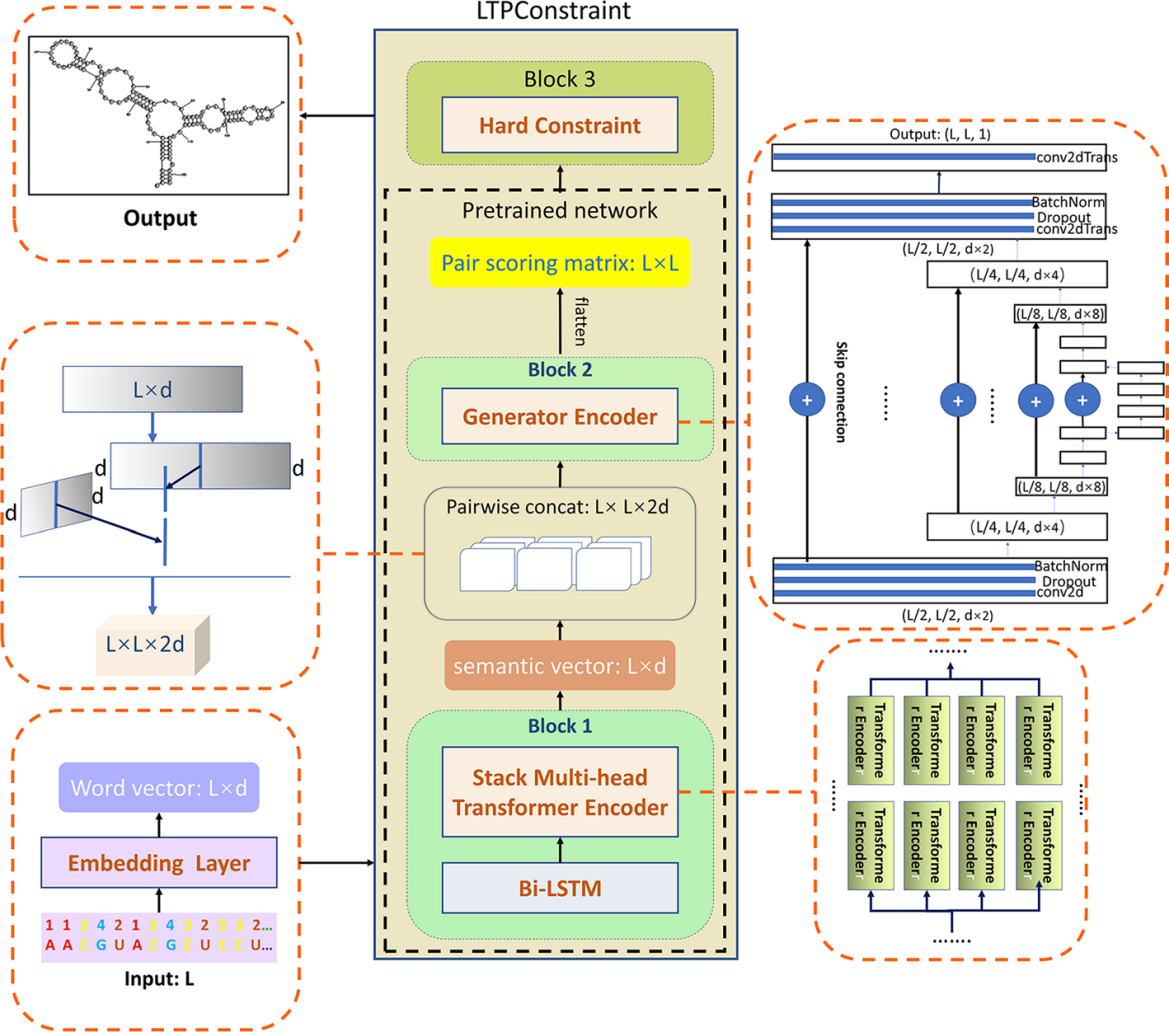
Architecture of LTPConstraint Network. Input module 1 after the sequence data goes through the Embedding layer. Pairwise concat is used for the output of module 1 and then input module 2. Until module 2, it is pretrained network. Finally, the preTrained network was modified with the hard constraint layer of module 3 to get the output

LTPConstraint uses a complex deep neural network to predict RNA secondary structure. The model’s input is the sequence after preprocessing, and the output is a $x\times x$ matrix, where *x* is the length of the sequence. The matrix values only from $\mathbf {S}=\{0,1\}$, the value 0 represents two bases do not match, the value 1 represents two base pairing. The whole network framework can be seen from the Fig. 5.

**Fig. 6:**
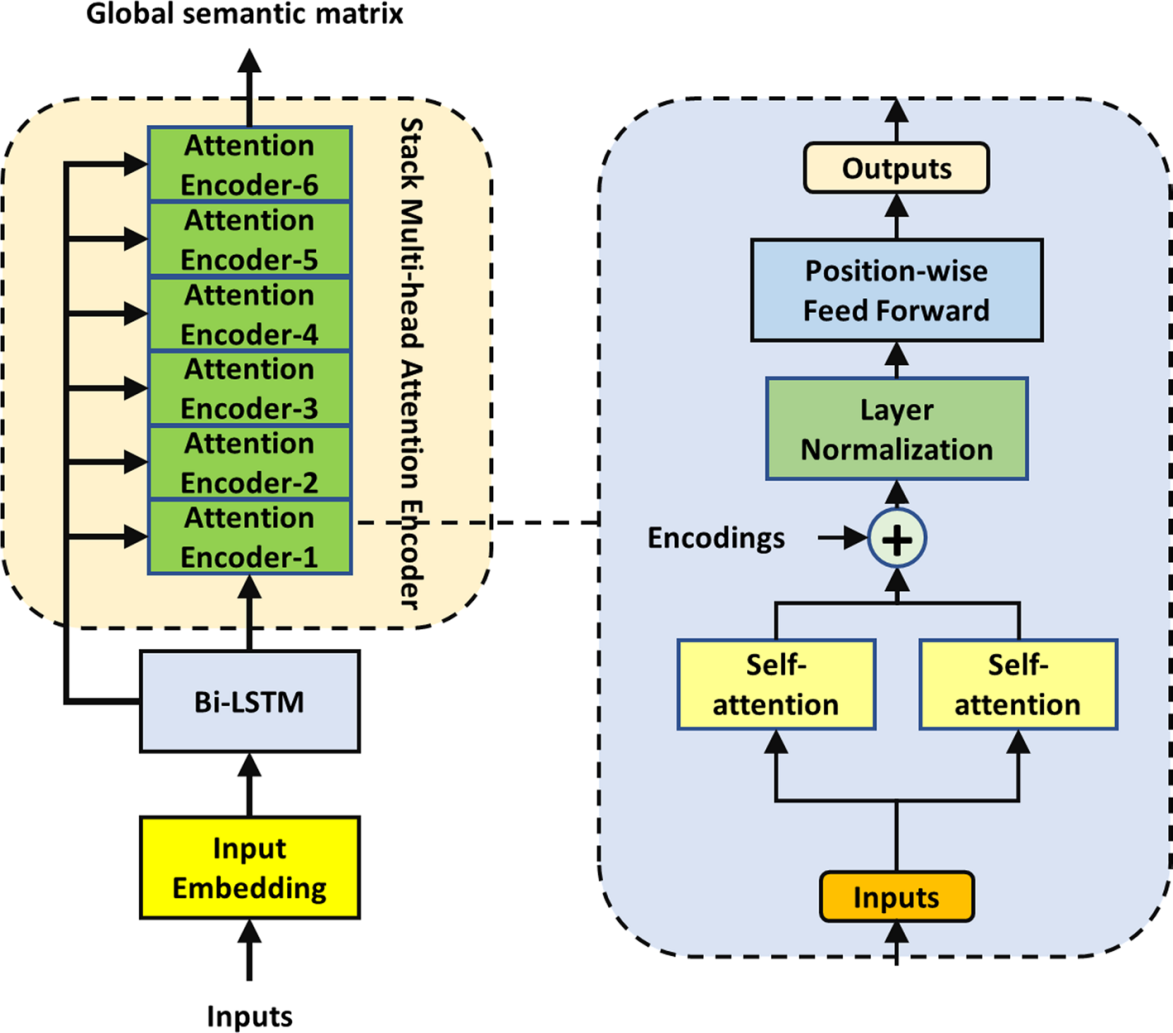
The structure of the global semantic extractor. This layer consists mainly of a Bi-LSTM layer and a 6-layer dual-headed Transformer encoder

The model is made up of three modules. The first module is a global semantic extraction module. The input of this module is a preprocessed sequence vector, which is converted to a word vector with channel 10 by an embedding layer. This step is different from the direct one-hot processing method of RNA sequence. The method of one-hot encoding gives the word vector a high-dimensional representation in an artificial way. However, a suitable representation of each base in the sequence is related to the distribution of input data and the structure of the model, so it is more appropriate for the model to learn the representation of the word vector directly from the data. Therefore, an embedding layer is used instead of one-hot. The global semantic extractor consists of two parts. The whole structure can be seen from the Fig. 6. The extractor starts with a single-layer bidirectional LSTM network, and then a Transformer Encoder. The Transformer Encoder contains six layers of 2-head self-attention module. This configuration of Transformer Encoder has been experimentally confirmed to achieve best cost-effective. This whole structure is to realize the complementarity of Bi-LSTM and Transformer. Bi-LSTM has good semantic extraction capability and implicit location information. Transformer Encoder is superior in semantic extraction and parallelism due to its computing structure. The global semantic extractor can take advantage of the two semantic extraction capabilities. Meanwhile, Transformer Encoder takes the output of Bi-LSTM in each timing sequence as input, which always implies the location information without manual input.

**Fig. 7:**
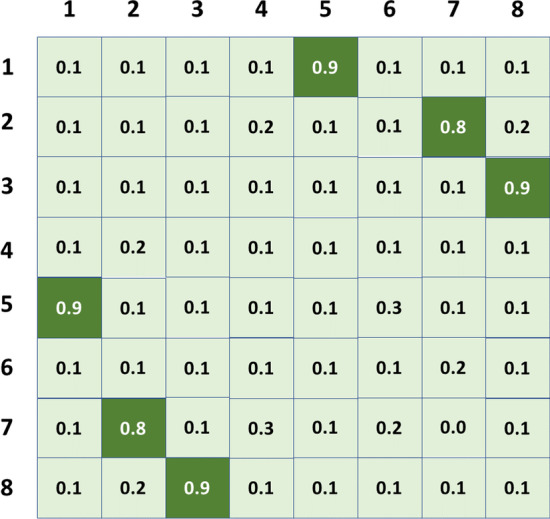
The structure of the scoring matrix. Light green represents the position that favors the score of negative cases, and dark green represents the position that favors the score of positive cases

The output of the global semantic extractor is a matrix of word vector. Then we need to fold the word vector. In detail, each word vector in the matrix is conbined with other word vector one by one to get a new 2-dimension matrix. it is similar to an adjacency matrix, and the difference is just that elements of each position are the splicing of two word vectors. The new matrix will become input of next module of our network. The next module will further refine these semantics to get a score for each pair which represents the probability that the model predicts pairs of each base, and this module is called the local feature extraction module. The output is like the Fig. 7. Input and output of this module has the characteristics that they have different data appearance but similar underlying structure coincidentally with the characteristics of image translation. However, we cannot apply the method of image translation directly. Generated adversarial network is used in image translation, but our output target is an adjacency matrix. Each element of this matrix values from 0 to 1. We know that one base can only be paired with another base, so the output matrix will be a quasi sparse matrix. We found in the experiment that generated adversarial network performs poorly when optimizing such targets. Because data with values close to one has insufficient impact on loss, The network optimized by generating adversarial network will output an adjacency matrix with all data of zero. Therefore, some experience in image translation can be used for reference in the problem of RNA secondary structure prediction. but the model structure and loss function need to be modified to adapt to the characteristics of this scenarios. Similarly, our label set is also an adjacency matrix $Y_{ij}$ like the Fig. 7, but the internal elements are only 0 and 1. 0 means that the base labeled i and the base labeled j are not paired, and 1 On the contrary.

**Fig. 8:**
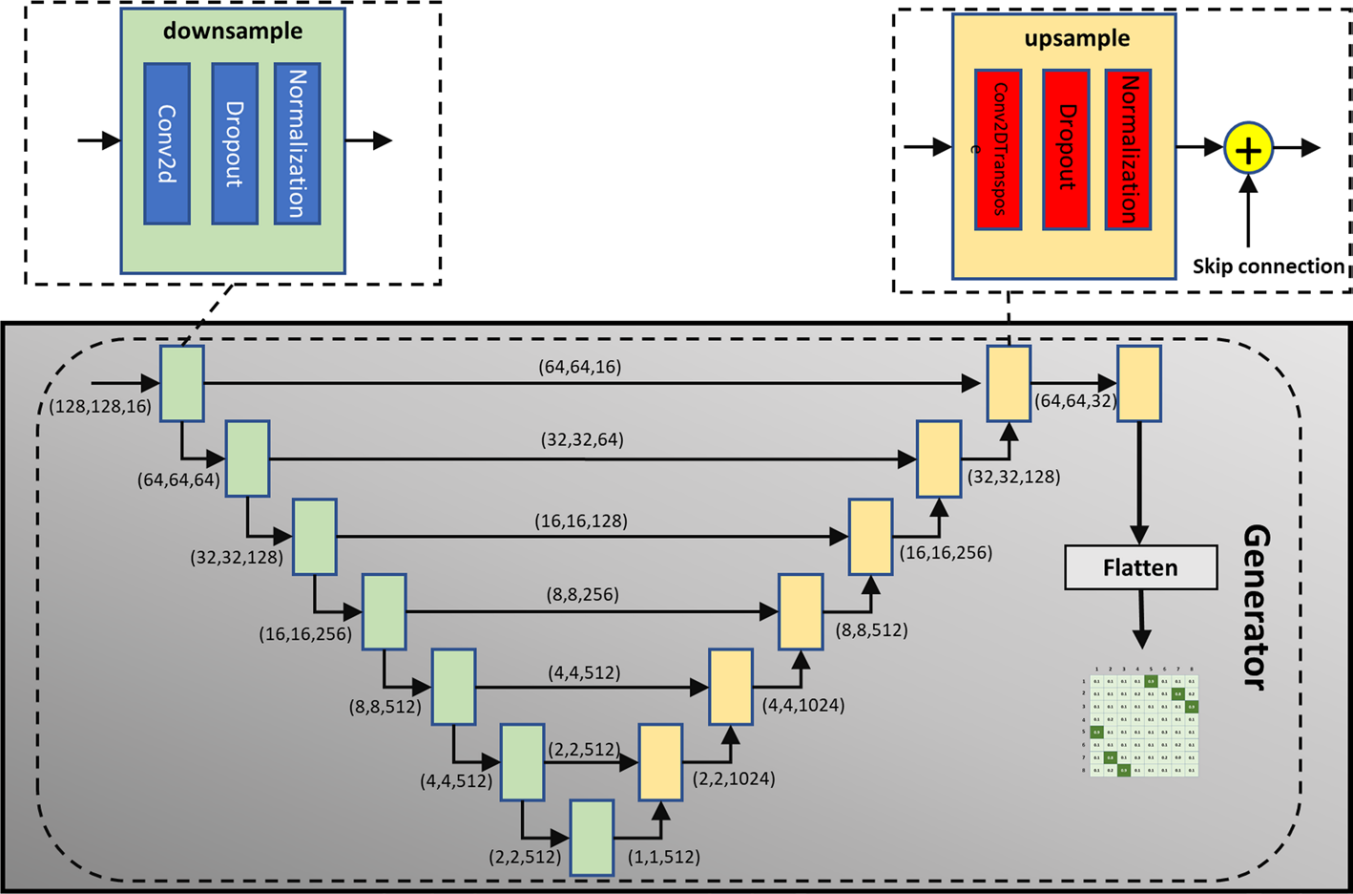
The structure of the generator network. This is a Unet structure. Each computing unit is a downsample or upsample structure. The structure given in the figure is designed for data with a sequence length of 128

In this paper, the generator of pix2pix model is used as the main body of the second module. The structure of generator can be seen in the Fig. 8.It uses the model structure of skip connection like Unet. output of layer i is directly added to layer n i, so that the input and output can share the underlying information, which helps extract the potential structural features between the input and output. However, the conditional discriminator in the pix2pix network is abandoned, and the characteristics of its antagonist network are transformed into a filtering network and placed in the third module. Meanwhile, specific loss functions are used to adapt to the characteristics of RNA secondary structure data. We have designed three kinds of loss functions and selected the one with the best performance as the final loss function after comparison. This experimental result is recorded in “Results” section. The design ideas of these three loss functions will be discussed below.

The label set used in this paper is an adjacency matrix values only from $\mathbf {S}=\{0,1\}$. The label means which base pairs up with each base in the actual RNA secondary structure. After statistical analysis, the number of value zero in the label accounts for 99.75% of the total, much larger than the value one. For the optimization goal of such extremely unbalanced data, both precision and recall should be considered when designing loss function.

1 $$\begin{aligned} p(x, y) = \frac{\langle x, y \rangle }{\langle x , y \rangle + \langle x , (1-y) \rangle } \end{aligned}$$

2 $$\begin{aligned} r(x, y) = \frac{\langle x , y \rangle }{\langle x , y\rangle + \langle (1-x) , y\rangle } \end{aligned}$$

In Eqs. (1) and (2), *x* represents the output of the model and the *y* represents the label, and the function $\langle \rangle$ means the matrix inner product. There are three types of loss functions that can be used, which are weighed cross-entropy loss, F1 loss, and AUC loss. The weight *pw* is added to the normal binary cross-entropy loss function to enlarge the impact of data with the value one on loss. If the *pw* value is too large, it will reduces the network’s ability to correct errors because the model attaches too much importance to the training of positive data and neglects the training of negative data, then false negative output will not get enough attention, resulting in premature network fitting. If *pw* value is too small, then the model will tend to be conservative, because the negative data account for the vast majority in the data set. The model will tend to predict all the data as negative examples, which is the conservative type caused by the data imbalance. In this paper, the binary search method is used to test and optimize *pw*, and finally achieved that the optimal value of *pw* is 256. The formula of weighed cross-entropy loss is Eq. (3)

3 $$\begin{aligned} l(x, y) = pw \cdot y \cdot -log(sigmoid(x)) + (1-y) \cdot -log(1-sigmoid(x)) \end{aligned}$$

In (3), *x* represents the output of the model and the *y* represents the label. Considering the precision and recall of the model, F1 scores can well reflect the situation of these two values and it is not affected by the balance of data. Therefore, this The distortion of F1 formula is very suitable to be the loss function of module 2, eventually becomes the Eq. (4).

4 $$\begin{aligned} F1(x, y) = -\frac{2 \cdot p(x, y) \cdot r(x, y)}{p(x, y) + r(x, y)} \end{aligned}$$

From the perspective of precision and recall, there is another way to construct loss function, to calculate the area under P-R curve, namely P-R AUC. Compared with ROC AUC, P-R AUC is much more useful in Binomial classification problems with unbalanced data distribution [48]. P-R curve models the sorting ability of positive and negative data using precision and recall. The sort ability embodies the careful consideration of learning under different tasks called expected generalization performance, and the AUC can reflect this ability. AUC value is the sum of the area under P-R curve. While the larger AUC value is, the better the performance of the model is [49]. Considering the calculation burden, this paper only takes 100 steps from 0 to 1 for the classification threshold, so that AUC is the sum of the areas of these 100 segments.

When calculating the AUC value, the model’s predicted value is divided into discrete values of positive and negative examples, bounded by the classification threshold. The predicted value greater than the threshold is positive example, and the predicted value less than the threshold is negative. However, the loss function needs to generate a gradient, so the decision process needs to be modified into a calculation process. Therefore, we use the continuous function relu to replace the discrete discriminant method. The formula for the entire AUC loss is Eq. (5), In Eq. (5), *x* means the recall value, and *y* means the precision value. We divide the circular which arc surrounded by the P-R curve, the P axis and the R axis into 100 parts according to the R axis. The height of each part is $\frac{1}{2}(y_i + y_{i+1})$, the width of each part is $x_{i+1} - x_i$, and the areas of the 100 parts are added up to get AUC.

5 $$\begin{aligned} AUC = -\frac{1}{2}\sum _{i=1}^{100}(x_{i+1} - x_i)\cdot (y_i + y_{i+1}) \end{aligned}$$

After testing, we used the Weighed-logistic as the loss function for our pre-trained network of module one and module two.

The third module of the model is a filter network because RNA secondary structure has its own structure rules, and the model needs to correct and fit the output accurately according to these rules. Its main task is to design a series of constraint rules, and then apply constraint rules to the model fitting. In [50], five kinds of hard constraints on RNA secondary structure were proposed. However, LTPConstraint needs to predict pseudoknot, so we need to remove some rules. The following four hard constraint rules were obtained:

1. Only canonical base pairing is allowed.
2. The base cannot pair with itself.
3. The paired two bases are at least four bases apart
4. Each base can only be paired with one base at most.

If we set the input sequence as $x=\{ x_1, x_2,\ldots,x_n\}$ and the canonical base pairing set as $\alpha =\{AU | UA\} \cup \{GC | CG\} \cup \{GU | CG\}$, the First three constraints can be expressed using the filter matrix *M* as follows (Table 1).

**Table 1:** Implementation of constraint rules

Constraint number	Implementation
Constraint 1	$if \{x_ix_j\} \subseteq \alpha , M_{ij}=M_{ji}=1$
Constraint 2	$if\ i=j, then\ M_{ij}=0$
Constraint 3	$\forall |i-j|< 4, M_{ij}=M_{ji}=0$

Three hard constraints that can be implemented using constraint matrice, corresponding to the first three of the four constraints listed in the text

The output of pre-trained model is a adjacency matrix $S_\theta (x)$ whose element values from 0 to 1. It’s a score of how likely each base is to pair. Module 3 will use the rules defined above to constrain the output of the upper network, and at the same time, we will also convert the score into a judgment of whether the reffered two bases matche. First, we define the output of the upper layer as $S_\theta (x)$ representing that it is the value obtained by the input through the previous network operation. Assuming that the output of each iteration is a new matrix *A*, then our optimization goal is the Eq. (7). Constant *b* represents the threshold value, part *f* of the formula expects the element of *A* to approach one when $S_\theta \ge b$, otherwise it expects the value of this position to be zero. The matrix *y* represents label. Part *g* of the formula is set to let A be as close to y as possible. The rest of the formula is an L1 regularization parameters. The real secondary structure label is a sparse matrix, so we add this item to make *A* as sparse as possible. $A1 \in R^N$ is a new matrix achieved by summing each row of *A*. $A1 \le 1$ represents the 4th constraint which mentioned above.

6 $$\begin{aligned} \begin{aligned} \max _{A\in R^{N\times N}}\underbrace{\langle S_\theta - b,A\rangle }_{\text {f}} + \underbrace{\langle y,A\rangle }_{\text {g}} - \eta \parallel A\parallel _1\quad \text {s.t.} \quad A1 \le 1 \end{aligned} \end{aligned}$$

This is a conditional extremum problem. So we introduce the Lagrange multiplier $\lambda \in R^N$ and generate the Lagrange function.

7 $$\begin{aligned} \begin{aligned} \min _{\lambda \ge 0}\max _{A\in R^{N\times N}}\underbrace{\langle S_\theta - b,A\rangle + \langle y,A\rangle - \langle \lambda , relu(A1-1) \rangle }_{\text {L}} - \eta \parallel A\parallel _1 \end{aligned} \end{aligned}$$

The corresponding duality problem is the Eq. (8).

8 $$\begin{aligned} \begin{aligned} \max _{A\in R^{N\times N}}\min _{\lambda \ge 0}\underbrace{\langle S_\theta - b,A\rangle + \langle y,A\rangle - \langle \lambda , relu(A1-1) \rangle }_{\text {L}} - \eta \parallel A\parallel _1 \end{aligned} \end{aligned}$$

We use gradient descent to solve the extreme point of each subproblems in the primal and dual problem. Therefore, it is necessary to find the derivation results of the L formula for $\lambda$ and *A* respectively, and then substitute them into the gradient descent formula to update the values of $\lambda$ and *A*. Then we can get the following equations.

9 $$\begin{aligned} \begin{aligned} A_{t+1}=A_t + \rho _{\alpha }^{t} \cdot A_t \circ M \circ (\frac{\partial L}{\partial A_t} + \frac{\partial L}{\partial A_t}^\mathrm {T}) \end{aligned} \end{aligned}$$

10 $$\begin{aligned} \begin{aligned} \frac{\partial L}{\partial A_t}=S_\theta - b + y - (\lambda \circ sign(A1 - 1))1^\mathrm {T} \end{aligned} \end{aligned}$$

11 $$\begin{aligned} \begin{aligned} \lambda _{t+1}=\lambda _{t}+ \rho _{\beta }^{t} \cdot relu(A1-1) \end{aligned} \end{aligned}$$

In the above formula, a learning rate $\rho <1$ which is a power relationship with the number of cycles *t* is introduced, which constitutes a self-regulating learning rate. As the number of cycles increases, the learning rate will continue to decrease. In Eq. (9,10,11), we also add the matrix *M* formed according to the three hard constraint rules mentioned above, and symmetric the gradient of each time.

In the subsequent iterations, *A* needs to be denoised every time. The method used is the hard threshold algorithm, which is equivalent to $$A_{ij}=\left\{ \begin{aligned} A_{ij}, \quad |A_{ij} |> \eta \cdot \rho _{\alpha }^{t} \\ 0, \quad |A_{ij} |\le \eta \cdot \rho _{\alpha }^{t} \end{aligned} \right.$$. We can get Eq. (12).

12 $$\begin{aligned} \begin{aligned} A_{t+1} = relu(|A_{t+1} |- \eta \cdot \rho _{\alpha }^{t}) \end{aligned} \end{aligned}$$

After a certain number of s rounds of iterations, we need to put hard constraints on the $A_s$ and symmetric it to get the optimized result matrix *A*.

13 $$\begin{aligned} \begin{aligned} A=\frac{1}{2}(A_s \circ M + (A_s \circ M)^\mathrm {T}) \end{aligned} \end{aligned}$$

After obtaining the output *A*, the loss function (4) is used to calculate the loss value and the Adam optimization function is used to optimize the network.

Finally, this paper will explain the process of transfer learning. When training the pre-trained model, we remove the third layer of the network structure. We train the pre-trained model using the Weighed-logisitc function as the loss function. The data used for training is the data of the Rfam database. Training the network containing Transformer requires constant fine-tuning of the hyperparameters, so we set the learning rate to be reduced when the difference between the loss values of two epochs is less than 0.0004. After getting the pre-trained model, We reload the network and parameters of the pretrained model and add the aforementioned module 3 to the top layer of the network, and train this new network using data from the target RNA family. We use different fine-tuning strategies for different RNA families. For families with low data volume or high similarity to the Rfam data set which was used for pre-training, we freeze all the layers outside the top layer and train. For families with large data volume and low similarity, we train the whole network after loading the parameters of the pre-trained model. In the comparative experiments, only the SPR database satisfies the characteristics of small data volume and high similarity with pre-training dataset. In order to ensure that the model can obtain the best prediction effect, we use the first strategy in the transfer learning of SPR database, The rest of the databases use the second strategy. After fine-tuning and transfer learning, a series of models for different families can be obtained.

### Data collection and processing

In this paper, Transfer learning is used to train the network. The basis of transfer learning is pre-trained model. In order to train a good pre-trained model, a data set with wide coverage and uniform distribution in the data domain is needed. RNAs can be divided into different families according to the sequence length, shape and function. RNA sequences which belong to the same family share many features. To train a pre-trained model that can be widely used in RNA secondary structure prediction, a database with a group of family containing various RNA sequence data is needed.

The Rfam database [51] is a collection of RNA families. As of Rfam v14.5, the database contains a total of 3940 RNA families, with a data volume of 43,273. It can meet the needs of the pre-trained model. The raw data in this paper came from two databases, bpRNA [52] and RNAStralign [53]. Firstly, the pre-trained model is trained using Rfam data. We can then use the pre-trained model to perform transfer learning on the data of a specific family that needs to be predicted, which is a process of deriving a model with a specific function from a generalized model.

The following describes the data processing methods. Firstly, according to the sequence length of the input data, two encoding lengths 128 and 512 are set to separate the data with the sequence length of no more than 128 from the other data between 128 and 512. We name them PT-128 and PT-512 respectively. Then, We need to de-redundant the data set. For the Rfam data that needs to be used for pre-trained model, this paper uses CD-HIT-EST [54] to remove redundant data in the whole 43,273 pieces of data. The homology rate is set to 80%. Then the data with more than 80% homology rate is removed. For the data required for transfer learning, we still use the CD-HIT-EST to remove redundant data in each family separately. Above is to separate the two types of data to remove redundancy, and then we need to merge the two data to remove redundancy again. Finally, In Rfam data, 30,249 sequence data with low redundancy were obtained. Among them, PT-128 contains 21,487 pieces. PT-512 contains 8762 pieces. The data volume of each database is visible in the Table 4

**Fig. 9:**
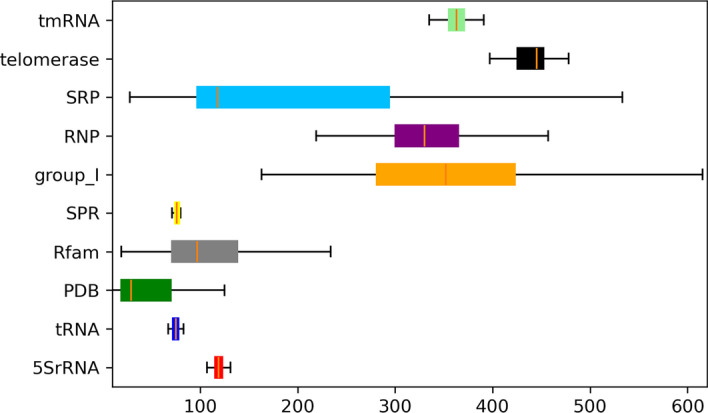
Length profiles of different sequences. The vertical axis of the box chart is the name of the database, and the horizontal axis is the sequence length, and the orange line on the box represents the median

The following will explain why we split the data into two encoding lengths. The length distribution of data from each family after processing can be seen in the Fig. 9.

It can be seen that the data of basically all databases are distributed into two sections between 0-128 and 128-512. Therefore, different pre-trained models should be used for transfer learning. In this paper, PT-128 data and PT-512 data will be used to train two pre-trained models aiming at the two kinds of sequence lengths respectively. This idea is based on such a scenario, if only one kind of pre-trained model is used, then the sequence with length less than 128 also needs to be processed into 512-length data for training. The effective length of input data will be less than 128, and the length of useless encodings will exceed 384. The ratio of the two will be less than 1:3. Valid sequences will be in an absolute minority. Then the model can consider the useless encodings as a valid sequence and affect the process of feature extraction. On the other hand, the 512 length of the training data is more expensive than 128 length of training data. The memory occupied by the length of the 512-length sequence is four times the memory size of the 128-length sequence, and the label set memory usage will be 16 times, the training network parameters will also be in the index level of 4 for growth. It can be seen from the Table 2, the number of 128-length sequences is far greater than that of 512-length sequences. If these 128-length sequences are processed into 512-length input data, it will cause huge computational force and storage resource waste. Therefore, this paper uses two kinds of encoding length and pre-trained models to train the data of two sections respectively.

**Table 2:** Sequence length tables for different databases

	Length	Samples
Rfam	19–3932	30,249
5SrRNA	104–132	10,788
tRNA	59–95	9245
PDB	4–2902	669
SPR	54–93	622
grpI	163–615	2135
RNP	189–486	466
SRP	28–533	959
telomerase	382–559	37
tmRNA	102–437	637

The length column represents the distribution of all sequences’ length in the database, and the samples column represents the number of sequences in the database

The Embedding layer in the network structure can independently learn the word vector of the base pair through the data. Therefore, we just need to maps the literal representation of the bases once and the input them into network. The mapping relationship is shown in the Table 3:

**Table 3:** Numeric coding of different bases

	Encoding
A	1
U	2
G	3
C	4
#	0

Before pre-training and transfer learning, this paper binds data and labels one by one and then randomly disrupts the order. 80% of the data is taken as the training set, 10% of the data is taken as the test set, and the remaining 10% is the verification set used in the experiment. The composition of the entire data set is shown in the Table 4:

**Table 4:** The composition of the entire data set

	Samples	Train	Test	Validate
Rfam_128	21487	17190	2149	2148
Rfam_512	8762	7010	876	876
5SrRNA	10788	8630	1079	1079
tRNA	9245	7396	925	924
PDB	669	535	67	67
SPR	622	498	62	62
grpI	2135	1708	214	213
RNP	466	373	47	46
SRP	959	767	96	96
telomerase	37	30	3	4
tmRNA	637	510	64	63

The column of samples represents the total number of a data set. The column train represents the data volume of the training set, and similarly test and validate represent the data volume of the test set and validation set

## Results

This paper is committed to using a novel end-to-end method to solve the problem of predicting RNA secondary structure. Roughly speaking, we use transfer learning method to train the whole network mentioned above and get model to predict RNA secondary structure. Now we will design comparative experiments to test the performance of our model.

In each experiment, the state of prediction for each base pairs can be divided into four situations: true positive, false positive, true negative and false negative. *TP*, *FP*, *TN* and *FN* were used to represent the number of samples corresponding to the four scenarios, and the confusion matrix of the classification results was shown in the Table 5.

**Table 5:** Classification confusion matrix for dichotomy problems

Label	Prediction	
	Positive	Negative
Positive	TP	FN
Negative	FP	TN

The row name represents the state of that position in the label set, Positive represents 1, and Negative represents 0. The column name represents the state of that position in the prediction results. TP and FP represent the predicted results are consistent with the real results. FP represents false positives, and FN represents false negatives

To better verify the model’s accuracy, the precision ratio P and recall ratio R were introduced to measure the model’s prediction ability.

14 $$\begin{aligned} P=\frac{TP}{TP+FP} \end{aligned}$$

15 $$\begin{aligned} R=\frac{TP}{TP+FN} \end{aligned}$$

However, recall rate and precision rate are mutually restricted. When recall rate is high, precision rate tends to be low. Similarly, when recall rate is low, precision rate tends to be high. If we need a win-win outcome, we need to introduce a measure *F*1.

16 $$\begin{aligned} F1=\frac{2\times P \times R}{P+R} \end{aligned}$$

When the value of *F*1 is high, it means that the values of *P* and *R* are both high. Therefore, in the experimental part of this paper, *P*, *R* and *F*1 will be used simultaneously to measure the model’s accuracy. All experimental data in this section are reserved to four decimal places.

### Experiment for the effect of different pre-training loss and constraint layer

In the section of method and materials, three loss functions are proposed for the fitting of the pre-trained model. In order to confirm which loss is most suitable for this topic, a comparative experiment should be designed for verification. Since this loss function is used to train the pre-trained model, this paper conducts tests on RFAM data, and the test results are as follows.

**Table 6:** Comparison of effects of models using different pre-training loss

Loss	Pre-train			Train		
	Precision	Recall	F1-score	Precision	Recall	F1-score
PRAUC-loss	0.0351	0.6177	0.0665	0.8599	0.7897	0.8233
Negative-F1	0.1564	0.0987	0.1210	0.3587	0.2107	0.2655
Weighed-logistic	0.0283	0.7559	0.0546	0.8963	0.8015	0.8462
No-pretraining	–	–	–	0.8907	0.5861	0.7070

In the pre-training phase, the hard constraint layer is removed from the network and different loss functions are used for training. In the training phase, we used different pre-trained models for transfer learning to obtain prediction models on the Rfam dataset. The loss function of training phase is Neagtive-F1 function

As can be seen from the Table 6, the results obtained by using Negative-F1 are significantly different from the other two functions. Judging from the performance of pre-training, the *P* value of the Negative F1 model was higher than the other two models, but the *R* value was far lower than the other two models, indicating that the prediction of this model contains too many *FP* samples. In addition, the other two models have achieved better performance during transfer learning process, but the performance of the negative-F1 model has not improved much. This is because the loss function used in transfer learning is still the negative-F1 function. The same loss function used in the two training process makes it impossible for training to get rid of the local optimum trapped in the pre-training process. As can be seen from the data in the table, in the training phase, the performance of the model pre-trained by Weighed-logistic function is slightly better than PRAUC-loss. In terms of operation overhead, since PRAUC-loss needs to calculate the partitioned area under the P-R curve for 90 times, the storage overhead and operation time are not as good as Weighed-logistic. In conclusion, using Weighed-logisitc function as the pre-training loss can achieve extremely high prediction accuracy and relatively small operation overhead, which is the best scheme for pre-training.

Comparing the pre-train and train(transfer learning) phase in the Table 6, it can be found that the addition of hard constraint layer can significantly improve the performance of the model. According to the data analysis, in the PRAUC model, the transfer learning model with the constraint layer which is also called module 3 increases by 1138% in the *F*1 score and 2350% in the *P*% value over the pre-trained model, and the *R* value is increased by 27.85%. In Negative-F1, the model with constraint layer improved by 119.4% in the *F*1 score, 129.3% in the *P* value, and 113.5% in the *R* value. In Weighed-logistic, the model with hard constraints was improved by 1138% in the *F*1 score, 3067% in the *P* value and 6.03% in the *R* value. To more clearly show how the hard constraint layer improves the prediction of the model, the Table 7 lists the values of *TP*, *FP*, *TN* and *FN* in the pre-training and training process for analysis.

**Table 7:** Comparison of confusion matrix value between pre-trained and trained model

Loss	Pre-train				Train			
	TP	FP	TN	FN	TP	FP	TN	FN
PRAUC-loss	98,045	2,691,310	66,601,770	60,662	125,331	20,411	69,272,660	33,376
Negative-F1	15,665	84,478	69,208,610	143,043	33,447	59,797	69,233,270	125,260
Weighed-logistic	119,968	4,113,699	65,179,376	38,740	127,198	14,718	69,278,350	31,510

Columns of Pre-train is the prediction confusion matrix of different pre-trained model, and columns of Train belongs to the model after transfer learning

As can be seen from the Table 7, between the two processes of pre-training and training, the number of *FP* samples decreased significantly. Except for the Negative-F1 model, the number of *FN* also decreased significantly, while the number of *TP* and *TN* increased slightly. This shows that the main function of the hard constraint layer is to screen out the wrong pairs. At the same time, since each bases can only have one pair, by constantly screening the legitimate pairs and retraining the whole network, some pairs that once fell into local minima and failed to generate the gradient are also corrected. The above experimental shows that the constraint layer plays a very necessary role in screening out errors and breaking local minima.

### Contrast experiments with other models

In this subsection, the validation set is used to test the ability of LTPConstrain against other good methods. The methods used for the comparison are CONTRAfold, LinearFold [55], ProbKnot [56], RNAfold and CycleFold [57]. The predicting results were converted into three values, *P*, *R* and *F*1, as shown in the Table 8:

**Table 8:** Contrast experiments with other models

Length	Family	LTPConstraint			CONTRAfold			LinearFold			ProbKnot			RNAfold			CycleFold		
		Precision	Recall	F1-score	Precision	Recall	F1-score	Precision	Recall	F1-score	Precision	Recall	F1-score	Precision	Recall	F1-score	Precision	Recall	F1-score
Encoding_128	Rfam	0.8599	0.7897	0.8233	0.5016	0.6186	0.5540	0.5429	0.5502	0.5465	0.4705	0.6118	0.5319	0.4598	0.5965	0.5193	0.2887	0.5329	0.3745
	5SrRNA	0.9857	0.9804	0.9831	0.6541	0.7337	0.6916	0.7330	0.7306	0.7318	0.5748	0.6036	0.5888	0.5716	0.6401	0.6039	0.3094	0.4984	0.3818
	tRNA	0.9985	0.9992	0.9988	0.7047	0.7787	0.7399	0.7445	0.7504	0.7474	0.6777	0.7691	0.7205	0.6659	0.7378	0.7000	0.3097	0.4695	0.3732
	PDB	0.6695	0.3050	0.4190	0.0180	0.0102	0.0130	0.0142	0.0071	0.0095	0.0228	0.0128	0.0164	0.0175	0.0105	0.0132	0.0324	0.0274	0.0297
	SPR	0.9929	0.9971	0.9950	0.6730	0.7496	0.7092	0.6990	0.6763	0.6875	0.6338	0.7319	0.6793	0.6355	0.7208	0.6755	0.3182	0.4865	0.3848
Encoding_512	grpl	0.8304	0.8894	0.8589	0.6589	0.6509	0.6549	0.6713	0.5557	0.6080	0.6124	0.6401	0.6259	0.6013	0.6383	0.6192	0.2068	0.1055	0.1397
	RNP	0.5334	0.7000	0.6054	0.5952	0.5998	0.5975	0.5335	0.4654	0.4972	0.5548	0.5383	0.5464	0.4956	0.5813	0.5350	0.2931	0.2008	0.2383
	SRP	0.7130	0.7378	0.7252	0.5731	0.6279	0.5992	0.6204	0.6099	0.6151	0.5693	0.6179	0.5926	0.5670	0.6234	0.5938	0.2621	0.3810	0.3105
	telomerase	0.3752	0.8728	0.5248	0.4327	0.6083	0.5057	0.4334	0.5670	0.4913	0.4026	0.5483	0.4643	0.3912	0.5572	0.4597	0.1193	0.2330	0.1578
	tmRNA	0.7550	0.8767	0.8113	0.4367	0.4592	0.4477	0.4208	0.3618	0.3891	0.3862	0.4350	0.4092	0.3828	0.4378	0.4085	0.1549	0.2107	0.1786

Six RNA secondary structure prediction models including LTPConstraint were tested using the processed validation set described in “Data collection and processing” section. Since LTPConstraint uses transfer learning, we need to use the pre-trained model. For the 5 families of Encoding_128 (see Table 4), LTPConstraint uses pre-trained model trained by using Rfam_128 data, while for the 5 families of Encoding_512, LTPConstraint uses the pre-trained model trained from Rfam_512 data

As can be seen from the Table 8, except for PDB database where the prediction effect of all models is poor, LTPConstraint’s performance on other databases is better than that of all other models, which reflects the accuracy of the LTPConstraint model. Cause the *F*1 value can reflect the level of *P* value and *R* value simultaneously, we extracted the *F*1 value for further analysis. Since the prediction results on the PDB database will affect the overall distribution, we remove the results on the PDB dataset. We use box diagram and histograms to show the distribution of *F*1 values and the mean values of *P*, *R* and *F*1.

**Fig. 10:**
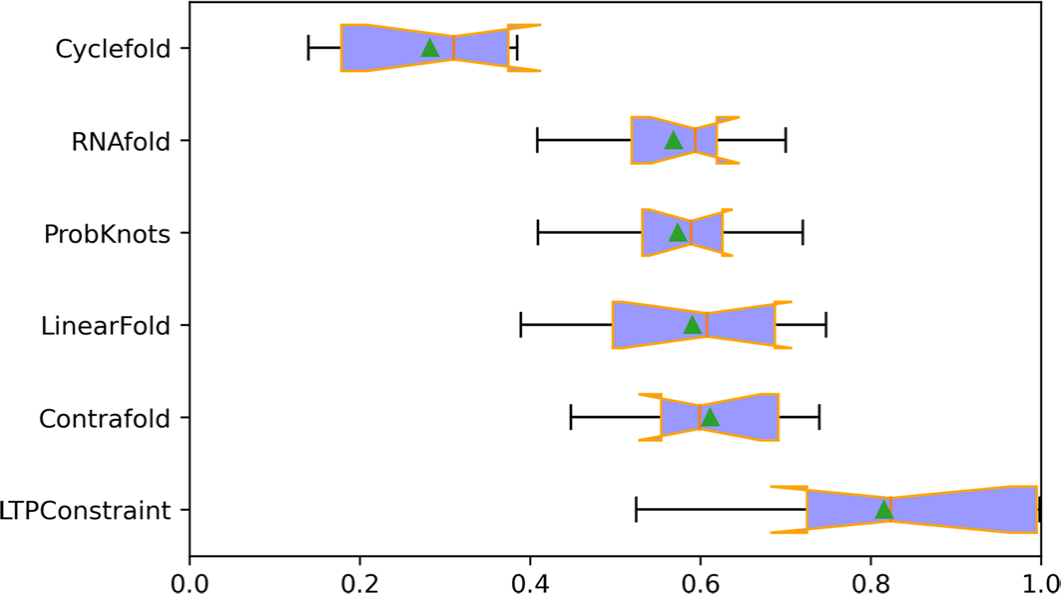
Box diagram showing all the *F*1 value predicted by the model. The orange line on the box represents the median, and the green triangle represents the average

**Fig. 11:**
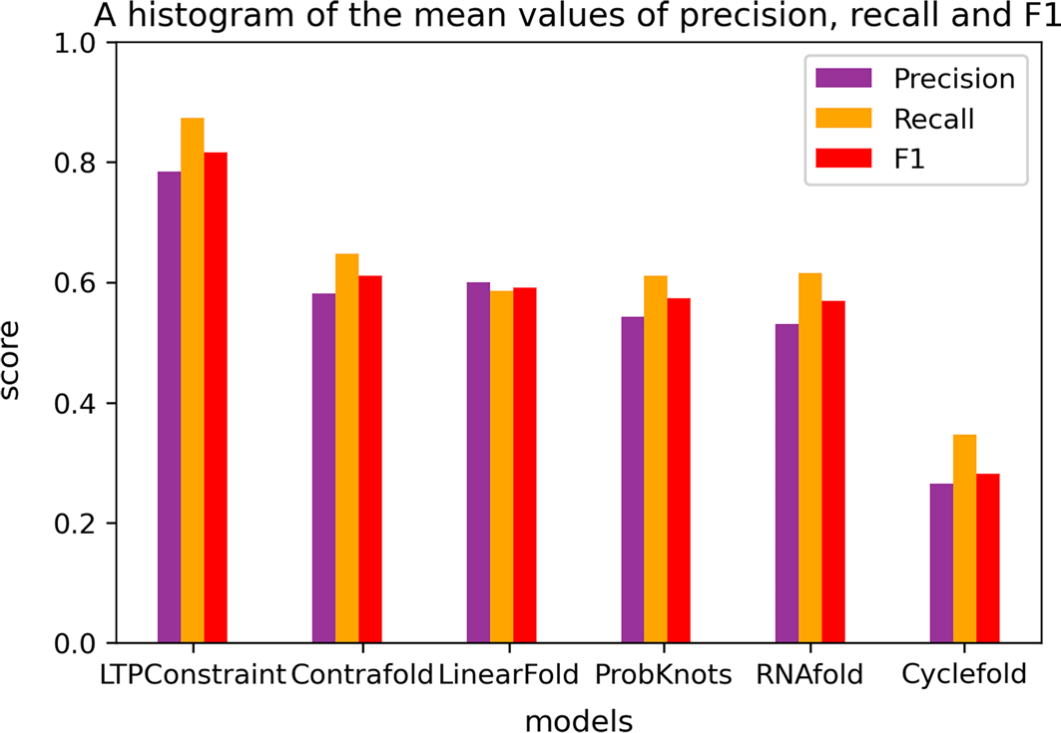
Histogram of the mean value of *F*1, *P* and *R*. The vertical axis of the histogram represents the value, and the horizontal axis is the name of each model. The purple column on the graph represents precision value, the orange column represents recall value, and the red column represents F1 value

It is clear from the Fig. 10 that LTPConstraint has stable and good performance. Comparing the stable areas of each model from the top quartile to the bottom quartile, it can be seen that LTPConstraint’s F1 value is closer to 1.0. The overall stability region of LTPConstraint has crossed chiefly the line of 0.8. In addition, the stability region is close to the upper edge line while the upper edge line basically coexists with 1.0. That is to say, the overall stability region is close to 1.0. This shows that the LTPConstraint model performs well in accuracy. Then we analyze the average prediction ability of each model from the Fig. 11. It can be clearly seen from the bar chart that LTPConstraint does better in the value of *P*, *R* and *F*1. By comparing with the best value of other methods, we find that LTPConstraint has achieved an increase of 0.1841 on the value *P* with an increase of 30.69% and 0.2259 on the value *R* with an increase of 34.89%, and 0.2046 on the value *F*1 with an increase of 33.48%. Through comparative experiments, we find that in the prediction of RNA sequences of different databases, the accuracy of LTPConstraint using transfer learning is obviously better than that of other models, and its performance is stable and can basically maintain the value *F*1 of 0.8 or above.

### Experiment on the role of transfer learning

This subsection will design a comparative experiment to test whether transfer learning really improves the performance of LTPConstraint. We used the control variable method to divide the experiment into two groups. The training set, test set and validation set of the two groups are identical. The first group uses the pre-trained model trained using Rfam data for transfer learning, while the second group does not carry out transfer learning and directly uses the database where the target data resides to train the whole network. In the whole experiment, the data set mentioned in subsection called data collection and processing is still used. Experimental results are shown in the Table 9.

**Table 9:** Table of comparative results to examine transfer learning

Length	Family	Transfer_learning				Non_transfer_learning			
		Precision	Recall	F1-score	ave-F1	Precision	Recall	F1-score	ave-F1
Encoding_128	5SrRNA	0.9857	0.9804	0.9831	0.8489	0.9777	0.8128	0.8876	0.6862
	tRNA	0.9985	0.9992	0.9988		0.8968	0.7873	0.8385	
	PDB	0.6695	0.3050	0.4190		0.2985	0.1600	0.2083	
	SPR	0.9929	0.9971	0.9950		0.8833	0.7489	0.8106	
Encoding_512	grpl	0.8304	0.8894	0.8589	0.7051	0.3002	0.0843	0.1317	0.1235
	RNP	0.5334	0.7000	0.6054		0.2432	0.1647	0.1964	
	SRP	0.7130	0.7378	0.7252		0.2236	0.3636	0.2769	
	telomerase	0.3752	0.8728	0.5248		0.0033	0.0072	0.0045	
	tmRNA	0.7550	0.8767	0.8113		0.0041	0.9996	0.0081	

In the group of transfer learning, two kinds of pre-trained model are still trained by using the data of Rfam_128 and Rfam_512. After the results of each group are available, two average F1 value is calculated for the families with two kinds of coding length

It can be seen from the Table 9 that the model with transfer learning has improved on the value of *P*, *R* and *F*1, especially in the data group with encoding length of 512, the improvement is more significant. In order to reflect the gap more clearly, the table also shows the average value of *F*1. According to the results, in the data with the encoding length of 128, the value *F*1 of the model using transfer learning is increased by 0.1673 on average, with a growth of 24.37% comparing with the model which don’t use transfer learning. While in the data with the encoding length of 512, it is increased by 0.5816 on average with a growth of 470.9%. It can be seen that transfer learning can significantly improve the accuracy of our model. However, the significance of transfer learning is obviously more than that. Why does a network perform well from the data with a short encoding length but not so well from the data with a encoding length of 512? This can be analyzed from the composition of data. The increase of encoding length will lead to the increase of features contained in the data. When the encoding length increases from 128 to 512, the amount of information increases by 16 times, and the features contained in the data may be more than 16 times. Therefore, more data should be input when training the data with the encoding length of 512 so that the training model can obtain the best effect of feature extraction, but in reality, the amount of data with the encoding length of 128 is about 5.1854:1 to that with the encoding length of 512. The effect of deep learning largely depends on the quantity and quality of data. If such a small amount of data is used for training, model is not able to learn enough. However, this problem can be solved well if we use transfer learning. The data with code length of 512 has a volume of 8762. After sufficient pre-training these data, the model has been mature in learning the shared features among data sets. Then transfer learning method is used to train for the target data. It can be seen in the table that a large improvement is produced using transfer learning. Therefore, Transfer learning is very necessary for training our network. It has the effect of improving accuracy and enabling the model to train some data sets with complex data but small data amount to partially overcome the dependence of deep learning on data.

### Experiment on predicting secondary structure containing pseudoknot

Since LTPConstraint uses pairing scores to indicate the pairing possibilities of two bases, the training of the network is not affected by the special spatial structure of pseudoknot, and the network is theoretically inherently suitable for predicting RNA secondary structure containing pseudoknot. To test this hypothesis, we need to design experiments. In the bpRNA database, there are many RNA sequences contain pseudoknot, and these sequences come from different families with different kinds of encoding length. Even if these data do not contain pseudoknot, it is still a difficult task to accurately predict these data’s secondary structure. Before describing the specific experiment, it is important to explain the data used. The raw data containing pseudoknot comes from bpRNA. Sequences are divided into two groups according to their length. Among these data, there are 2146 sequences with the length under 128, while there are 3011 medium-length sequences with the length between 128 and 512. The segmentation ratio of 0.8:0.1:0.1 is still adopted. This experiment will use such dataset to test the LTPConstraint model to see if the accuracy of LTPConstraint will be significantly affected in the presence of pseudoknot. Also, some good models that can predict pseudoknot will also predict the same data. We compare the results between LTPConstraint and other models to see if the LTPConstraint model has the ability to predict pseudoknot. There are very few models that can predict pseudoknot and two of the most excellent ones are selected and compared with LTPConstraint. They are ProbKnot and Knotty [58]. The results are shown in the Table 10.

**Table 10:** Comparative test results of predicting sequence with pseudoknot

Model	Pseudoknot_128			Pseudoknot_512		
	Precision	Recall	F1-score	Precision	Recall	F1-score
LTPConstraint	0.9314	0.8769	0.9034	0.7477	0.7151	0.7310
ProbKnot	0.5305	0.5522	0.5411	0.3772	0.4136	0.3946
Knotty	0.5317	0.6708	0.5932	0.3290	0.3886	0.3563

In the training of LTPConstraint, two kinds of pre-trained model are still trained by using the data of Rfam_128 and Rfam_512

As seen from the Table 10, LTPConstraint’s accuracy improves by more than 50% over the other two models, which is a significant improvement. Moreover, compared to the value *F*1 obtained from the data without pseudoknot (Table 9), the LTPConstraint’s value *F*1 obtained from the data containing pseudoknot is even higher than the average. It can be seen that pseudoknot have no significant effect on LTPContraint’s results, confirming that LTPConstraint does have solid ability to predict structure containing pseudoknot.

## Discussion

When constructing the network, this paper uses a variety of neural network structure so that they can make up for their own shortcomings. The combined LTPConstraint network can give full play to the advantages of each substructure and improve the prediction accuracy. However, too much substructure causes the LTPConstraint network to be too large, which requires a large amount of memory resources during training. At the same time, the network transforms the sequence into an adjacency matrix, which leads to the two-dimensional labeling and the further expansion of the consumption of video memory, memory and computing resources, thus doubling the cost of training as the sequence is longer. In this paper, the limit encoding length is 512. For longer sequences, the training equipment used in this paper cannot meet the training requirements, so the training cost will affect the promotion of the LTPConstraint model. In addition, LTPConstraint’s dependence on the amount of data increases as sequence length increases, however, the number of long sequences is significantly less than the number of short sequences, which can cause LTPConstraint accuracy to slip when predicting medium and long sequences. At the same time, using transfer learning leads to the requirement of high quality data, but in the medium and long sequence under the condition of shortage, to ensure the data fields abundance has become a difficult task. This problem can be solved in the future if better databases provide more high-quality medium and long sequences.

## Conclusions

This paper constructs an end-to-end prediction model of RNA secondary structure LTPConstraint. The network has a variety of substructure, and different substructure cooperates with each other and complements each other, making up three modules of LTPConstraint network. The first module is composed of Bi-LSTM and Transformer Encoder to extract the deep semantic and matching information of the base sequence. In the second part, the local pairing information is transformed by a generator network, and the scoring matrix of each base pairing is generated. Then, the output in the form of an adjacency matrix is obtained by the modification and evolution of the hard constraint layer in the third module. We divided all the sequences into 128 and 512 levels according to the sequence length, and the data set obtained through careful selection was used for pre-training. Based on the pre-trained model, we use fine-tuning strategies to train models for the data set from different families, which reduced the training cost and improved the prediction accuracy of the model. That is the process of transfer learning. Through comparative experiments, we found that the use of appropriate loss function for pre-trained model can improve the effect of training. At the same time, we use the transfer learning method to greatly improve the accuracy of LTPConstraint in other RNA families that lack sufficient data. We compared the LTPConstraint model using transfer learning with other good models, and the results showed that LTPConstraint is better than other models in terms of accuracy and stability. On the premise of ensuring accuracy, the method used in this paper also partially overcomes the problem of deep learning’s dependence on data volume. Although LTPConstraint does good in RNA secondary structure prediction, we still think that our work is just a supplement of deep learning method for the problem of predicting RNA secondary structure. Simultaneously, the model still has many problems such as the high cost of training, the prediction accuracy is reduced due to the insufficient number of long sequences. In future work, we will optimize the compatibility of LTPConstraint for predicting long sequences while reducing the computational resources used by the model. We will also make the model more user-friendly and easier to generalize. We will apply this secondary structure prediction method to biological experiments, so as to provide biologists with more accurate reference and make contributions to the field of life science.

## Data Availability

The datasets generated and analysed during the current study are available in the LTPConstraint repository, https://github.com/jluF/LTPConstraint.git.
